# Osseous and Nonosseous Bone Scan Findings in Liver Transplant Candidates with end-stage Chronic Liver Disease

**DOI:** 10.4274/Mirt.29292

**Published:** 2013-08-01

**Authors:** Seval Erhamamcı, Ayşe Aktaş, Tatiana Bahçeci, Kevser Kavak

**Affiliations:** 1 Başkent University Faculty of Medicine, Department of Nuclear Medicine, Ankara, Turkey

**Keywords:** scintigraphy, liver diseases, secondary hypertrophic osteoarthropathy

## Abstract

**Objective:** End-stage chronic liver disease (CLD) adversely affects the function of multiple organ systems including the skeletal system. The aim of this study was to assess osseous and nonosseous bone scintigraphy (BS) findings in liver transplant (LT) candidates with end-stage CLD.

**Methods:** We retrospectively evaluated BS findings in 50 consecutive patients with end-stage CLD who were undergoing preoperative assessment for LT from January 2006 to December 2011. All the patients were analyzed with respect to clinical and laboratory parameters, and BS findings. Scintigrams were visually assessed for the presence of osseous and nonosseous abnormalities. Osseous abnormalities were classified as those indicating bone metabolism changes or metastatic bone disease. Typical scintigraphic findings denoting to changes in bone metabolism were the presence of decreased osseous uptake, increased periarticular uptake, asymmetrical or unusual uptake patterns. Nonosseous findings were classified according to the degree of soft-tissue uptake as mild and severe.

**Results:** The group consisted of 46 adult and 4 adolescent patients. All adolescent patients had normal skeletal accumulation with growth plate uptake and one had mildly increased renal cortical activity. A total of 46 adult patients had one or more of the following osseous findings: generalized decrease in osseous uptake (n=4, 8.7%); bilateral decrease in lower extremity uptake (n=26, 56.5%); symmetrically increased periarticular uptake (n=26, 56.5%); bilateral cortical/periosteal increased uptake in the lower extremity indicating hepatic hypertrophic osteoarthropathy (HOA) (n=8, 17.4%); bilateral increased sacroiliac activity (n=16, 34.8%); sacral activity (n=10, 21.7%), coccygeal activity (n=2, 4.3%), focally increased uptake suggestive of metastases (n=5, 10.9%). Three rib metastases appeared to be linear. Nonosseous findings observed in adult patients were mild diffuse liver uptake (n=4, 8.7%) and bilateral diffuse mild or severe degree of renal cortical uptake (n=20, 43.5%). There was a statistically significant difference in serum creatinine values between mild and severe renal uptake groups (p<0.05). There was also statistically significant difference in serum BUN and creatinine values between patients with severe degree of renal uptake and without renal uptake (p<0.05).

**Conclusion:** The results of the current study has shown that adolescent LT candidates with end-stage CLD had no osseous abnormality on BS. However, all of adult patients exhibited one or one more osseous abnormalities. Typical scintigraphic findings denoting to abnormalities in bone metabolism were generalized decreased osseous uptake, decreased lower extremity osseous uptake, increased periarticular uptake, increased cortical/periosteal uptake indicating hepatic HOA, and other unusual uptake patterns. Hepatocellular carcinoma metastases may present itself as rib metastases linear in pattern. Soft-tissue uptake in the form of diffuse bilateral mild or severe degree of renal uptake and less commonly mild diffuse liver uptake can be observed. Increased renal uptake may be an early marker of renal dysfuntion or a predictor of hepatorenal syndrome.

**Conflict of interest:**None declared.

## INTRODUCTION

Abnormalities in bone metabolism observed in chronic liver disease (CLD) are referred to as hepatic osteodystrophy (HO), which includes osteoporosis and, less frequently, osteomalacia. The prevalence of bone disease has been reported in previous studies to range between 12% and 55% among patients with liver cirrhosis (1,2,3,4). Following liver transplantation (LT), the fracture risk is further increased due to the use of high steroid doses and prolonged immobility, particularly when complications arise after surgery. The fracture rates after LT have been reported to be 15% to 34% (1). Osteoarthritic changes due to osteopenia and rheumatic complications were also reported following transplantation (5). Hepatic hypertrophic osteoarthropathy (HOA) has been documented in case reports (6,7,8,9,10). In addition to metabolic bone disease, metastatic bone disease was encountered following long periods of cirrhosis giving rise to the high incidence of hepatocellular carcinoma (HCC) (11,12). Bone disease in patients with CLD have a significant impact on morbidity causing fractures that may result in chronic pain, long-lasting immobility, and deformity. Therefore, routine bone status assessment should be performed in all patients with CLD in order to minimize the bone loss and decrease the fracture risk after transplantation. 

Bone scintigraphy (BS) with Technetium-99m hydroxy diphosphonate (^99m^Tc HDP) is usually performed to evaluate a wide variety of skeletal abnormalities, including metabolic and metastatic bone diseases (12,13,14). Although BS is a test primarily concerned with skeletal abnormalities, important nonosseous findings can occasionally present themselves on the images (15,16,17,18), because renal function and metabolic status of the patient strongly affect the scan appearance (15). The recognition of specific conditions with soft-tissue uptake of bone-seeking tracers is important as it greatly enhances the diagnostic value of the study. 

In the literature, there are a limited number of reports about the osseous abnormalities indicating bone metabolism changes or metastatic bone disease on BS during follow-up in patients with CLD awaiting LT (11,14). In addition, there are a few case reports about the soft-tissue uptake on BS in cirrhotic patients. The aim of this study was to assess osseous and nonosseous BS findings in LT candidates with end-stage CLD. 

## MATERIALS AND METHODS

**Patients**

This retrospective analysis consisted of 50 (46 adults and 4 adolescents) consecutive LT candidates who underwent ^99m^Tc HDP BS from January 2006 to December 2011 in our instution. Bone scan with ^99m^Tc HDP was performed within one month prior to LT. The diagnosis of CLD and HCC was made on the basis of liver biopsy and/or clinical, laboratory and radiologic findings. This study was conducted by medically qualified personel in strict accordance with the guidelines of the Baskent University Medical Faculty Institutional Review Board following the Tenets of the Declaration of Helsinki for retrospective analyses.

**Bone Scintigraphy**

Bone scintigraphy were obtained approximately 3 h after the intravenous injection of 740 MBq (20 mCi) ^99m^Tc HDP for adults. In adolescents, the dose of ^99m^Tc HDP was adjusted for body weight. Whole-body images (anterior and posterior views, scan speed 15 cm/min, matrix 256x1024) were obtained with a double-headed gamma camera equipped with low-energy high-resolution parallel hole collimators (Siemens, e.cam, Erlangen, Germany). Anterior and posterior spot views of the body were obtained using 256×256 matrix for 500.000 counts. 

**Image Interpretation**

All bone scintigraphies were visually assessed by two experienced nuclear medicine physicians without the knowledge of the patient’s clinical information. Scintigrams were evaluated for the presence of osseous and nonosseous abnormalities. Osseous abnormalities were classified as indicating bone metabolism changes or metastatic bone disease. Typical scintigraphic findings denoting to bone metabolism abnormalities were the presence of decreased osseous uptake, increased periarticular uptake, increased cortical/periosteal uptake suggesting hepatic HOA and unusual uptake patterns including sacroiliac, sacral and coccygeal activity.

Nonosseous findings were also classified according to the degree of soft-tissue uptake. The level of renal uptake of ^99m^Tc HDP was assessed in the posterior position and graded as mild: accumulation equal to or higher than lumbar spine; severe: accumulation significantly higher than lumbar spine.

^99m^Tc HDP uptake of liver was compared against the background activity and close to background activity described as mild level. 

**Biochemical Measurements**

For biochemical examinations, calcium (Ca), phosphorus (P), alkaline phosphatase (ALP), aspartate aminotransferase (AST), alanine aminotransferase (ALT), γ-glutamyl transferase (GGT), blood urea nitrogen (BUN), and creatinine values were measured in venous blood samples following fasting for one night.

**Statistical Analysis**

All data were expressed as mean±2SD. Student t-tests were used to analyseanalyze all mean values and to compare differences between the groups. The Pearson test was used for correlation. All statistical analyses were performed using the statistical package for the social sciences software (SPSS, version 15.0; SPSS Inc, Chicago, IL, USA) for Windows, and a P value of less than 0.05 was considered statistically significant. 

## RESULTS

Patient CharacteristicsThe detailed patient characteristics for this study are presented in Table 1. The group consisted of 46 adult (43 male and three female; mean age: 56.80±8.15 years; range 36-80 years) and 4 adolescent patients (2 male and 2 female; mean age: 15.0±0.81 years; range 14-16 years).

In adolescent patients, etiology of liver disease was co-infection of HBV and HDV in 1, cryptogenic in 2 and Wilson diseases in 1. Three of 4 adolescent patients had HCC and 4 underwent LT. In adult patients, etiology of liver disease was viral in 42 (91.3%), alcoholism in 1 (2.2%), and cryptogenic in 3 (6.5%). Hepatitis B was diagnosed in 23 (50%), hepatitis C in 16 (34.8%), co-infection of hepatitis B and hepatitis C in 1 (2.2%), and co-infection of hepatitis B and hepatitis D in 2 (4.3%). Forty-five (97.8%) of 46 patients had HCC; 8 (17.4%) underwent LT; 2 (4.3 %) had only one kidney, and 7 (15.2 %) died from complications due to liver failure during follow-up. None of the patients reported a history of bone fractures during the scan. Mild-severe degree of ascites was present in all (50) patients at the time of the scan. 

Table 2 and 3 lists the results of the laboratory tests. The serum levels of liver enzymes (AST, ALT, GGT, and ALP) were higher in the adults. Out of 4 adolescent patients, 3 had high BUN levels. Out of 46 adult patients, 18 had high BUN levels. Twelve patients with high BUN level had also high creatinine level.

**Figure 1:**
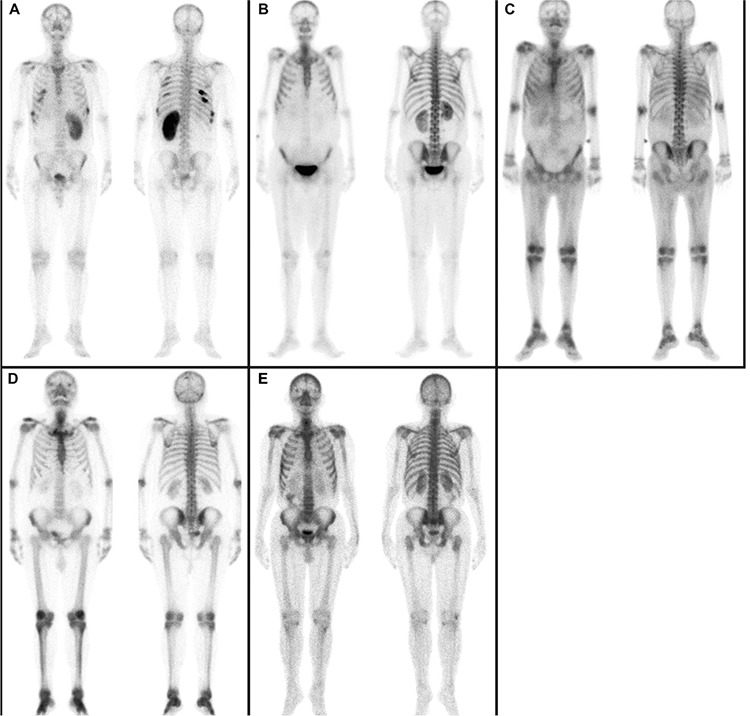
Whole-body bone scintigrams in an adult male patients with end-stage CLD together with HCC resulting from viral etiology. (A) There is generalizeddecreased skeletal uptake, severely increased diffuse renal uptake in a kidney having compensatory hypertrophy in the absence of controlateral kidney,and rib metastasis that appear to be linear, (B) bilateral decrease in lower extremity uptake and severely increased bilateral renal cortical uptake, (C) generalbilaterally increased periarticular uptake, lower long bone activity and mild diffuse hepatic uptake, (D) hepatic HOA leading to bilateral increase in lowerextremity distal long bone cortical uptake together with periarticular uptake, (E) bilateral sacroiliac and sacral activity, bilateral lower limb uptake and diffusemild increase in renal uptake.

Scintigraphic results are reported in Table 4. All adolescent patients had normal skeletal accumulation with growth plate uptake and 1 had mild increased renal cortical activity. Adult patients had one or more of the following osseous findings: 

1. Generalised clearly decreased osseous uptake (n=4, 8.7%) (Figure 1A). This finding was accompanied with severe increased renal uptake.

2. Bilateral decreased osseous uptake in the lower extremity (n=26, 56.5%) (Figure 1B). 

3. Symmetrically increased periarticular uptake (n=26, 56.5%) (Figure 1C). This was more pronounced in the knee and/or ankle. 

4. Bilateral cortical/periosteal increased uptake in the lower extremity of long bones (tibial and/or femoral) indicating hepatic HOA (n=8, 17.4%) (Figure 1D). This finding was also associated with periarticular uptake. 

5. Other unusual uptake patterns were bilateral increased sacroiliac activity (n=16, 34.8%) (Figure 1E); sacral activity (n=10, 21.7%) (Figure 1E); coccyx activity (n=2, 4.3%). 

6. Focally increased uptake suggestive of metastases (n=5, 10.9%). Out of 5 patients, 3 had only rib metastasis that appeared to be linear, 1 had only thoracic vertebral, and 1 had rib together with thoracic vertebral metastases. These bone lesions were confirmed by plain radiographs and computed tomography. 

Nonosseous finding observed in adult patients were mild diffuse liver uptake (n=4, 8.7%) (Figure 1C) and bilateral diffuse increased renal cortical uptake (n=20, 43.5%) (Figure 1A, B, E). 

In 20 adult patients with bilateral diffuse increased renal cortical uptake, the mean serum BUN level was high (29.00±27.23 mg/dL). Out of these 20 patients, 10 had mild and 10 had severe degree of renal uptake. The individual data showed high serum BUN levels in 10 (50%) patients including 4 with mild and 6 with severe degree of renal uptake. Of these 6 patients with severe degree of renal uptake, 5 also showed high serum creatinine levels. There was a statistically significant difference in serum creatinine values between mild and severe renal uptake groups (p<0.05) (Table 4). There was also statistically significant difference in serum BUN and creatinine values between patients with severe degree of renal uptake and without renal uptake (p<0.05). 

There was a positive correlation between increased renal uptake and both generalized decreased uptake and decreased lower extremity uptake in adult patients (p<0.05, p<0.001, r=0.35 and r=0.50 recpectively). Among 26 patients with decresed lower extremity uptake, 17 (65.4%) had increased renal uptake. Out of these 17 patients, 9 (53%) had severe and 8 had mild degree of renal uptake.

## DISCUSSION

Since liver plays an important role in bone metabolism, skeletal abnormalities in cases of deteriorated hepatic function is inevitable. The pathogenesis of bone disease in patients with CLD is thought to be multifactorial, with low vitamin D levels, immobility, hypogonadism, and reduced intestinal calcium absorption all being possible contributory factors. In addition, direct detrimental effects of alcohol, iron, and corticosteroids in patients with autoimmune liver disease have been implicated. The molecular mechanisms have not been elucidated; however, it is possible that circulating cytokines and growth factors involved in liver regeneration and repair may also modulate bone turnover (1,2,3,4).

Radionuclide imaging studies reflect bone blood flow and osteoblast activity to assess regional changes in bone metabolism. Many disease states, including osteoporosis, osteoarthritis, and bone neoplasms, result in disturbed bone perfusion. Bone scintigraphy has been acknowledged as a sensitive method for early detection and assessment of metabolic bone disease (13,14). Bone scintigraphy has also been shown to be a detector of HO (14). In this retrospective study, scintigraphic patterns of osseous and nonosseous changes related to CLD were presented.

In the present study, we examined adult population of mixed etiology. The majority of patients (91.3%) were affected by viral cirrhosis. Females were a minority of the patients in the study group. The mean age of patients was 56.8 years. None of our patients complained of clinical symptoms or reported a history of bone fractures. Generalizsed low skeletal uptake and bilateral decreased lower extremity uptake was present in 8.7% and 56.5% of adults, respectively. Generalized decreased uptake was accompanied with increased renal uptake, 65.4% (17/26) patients with decreased lower extremity uptake had also increased renal uptake and 53% (9/17) of them had severe degree. Generalizsed decreased skeletal uptake together with increased renal uptake have previously been described in a number of causes such as congestive heart failure, elevated serum aluminum levels, iron overload, osteoporosis, thalassemia major, and advanced renal osteodystrophy and hepatorenal syndrome (19,20,21). There is also a report on bilateral lower limb uptake of bone scanning agents (22). The degree of radiotracer uptake depends primarily on two factors: blood flow and, perhaps more importantly, the rate of new bone formation. Decreased blood flow in patients with advanced cirrhosis causes an alteration in the amount of delivered radioactivity and is associated with reduced accumulation in the skeleton. Bone marrow hyperplasia in chronic hepatocellular failure, loss of trabeculae, and cortical thinning with consequent loss of bone mass might be the causes of decreased bone uptake of the tracer (20). In addition, this finding can be due to osteoporosis or increased distance between gamma camera and lower limbs on whole body scanning in patients with severe ascites. 

In this study, bilateral increased periarticular uptake was observed in 56.5% of adults. It has been demostrated that osteoarticular complications result from the rise of immunosuppressive drugs in the posttransplant period and may also be favoured by a wide range of preexisting liver diseases (5). In a study, remarkable improvement in arthritis have been shown following LT (23). Both growth hormone excess and deficiency have been depicted to lead to changes in the incidence of osteoarthritis. A previous report also suggests that increased periarticular uptake can be a metabolic response to pharmacological agents (24). Hepatic HOA was present in 17.4% of adults and all of them had also periarticular uptake. Hypertrophic osteoarthropathy is rare, characterizsed by periosteal reactions affecting the shafts of long bones, frequently associated with periarthritis. There have been cases of HOA secondary to biliary atresia reported in the literature. Hypertrophic osteoarthropathy with associated arthritis was described in patients with hepatic failure and transplantation (23). It has been emphasized that when HOA occurs in the setting of liver disease it usually heralds a worsening of hepatic function (6). The pathogenesis of HOA is unknown. Hormonal, circulatory, and neurogenic factors have been implicated. It is thought that a growth factor mediated effect is probably involved leading to elevation of the periosteum, new bone deposition and oedema of the surrounding tissues. This factor may accumulate as a result of impaired hepatic clearance or may be produced in excess by the liver as part of its response to disease. 

Other unusual uptake patterns denoting to bone metabolic abnormalities were increased sacroiliac, sacral and coccygeal activity that was observed in 34.8%, 21.7%, 4.3% of our adults, respectively. Sacroiliac, sacral and coccygeal activity can be due to the pressure effect in patients with severe ascites. Sacral and coccygeal activity may result from occult insufficiency fractures, because our patients population had potential risk factors such as dietary deficiencies, osteoporosis and immobilization. However, this finding was not confirmed with other imaging modalities, because of the retrospective nature of the study. Occult insufficiency fractures of the sacrum resulting from osteoporosis have been reported in postmenopausal women, elderly and in patients with advanced CLD (25,26). 

Bone metastases for in HCC patients have been encountered more frequently during the past decade. Fukutomi et al. have reported a high bone metastasis rate of 13% in HCC patients (11). In our study, metastatic bone lesions were observed in 11.1% (5/45) of adults with HCC, in accordance with Fukutomi et al. The most commonly reported sites for bone metastases were the vertebrae and rib (11,12), as is the case in the current study. In addition, rib metastases appeared to be linear. 

Another focused issue of the current study was to evaluate soft-tissue uptake in patients with CLD awaiting LT. In the literature, more specific information about the increased renal parenchymal uptake and/or liver uptake in patients with cirrhosis is usually found in case reports. It is also difficult to ascertain the exact prevalence of soft-tissue uptake because the BS study was not routinely performed for all CLD patients. Soft-tissue accumulation of bone-seeking agents can occur in cirrhosis and cannot be explained by a single mechanism and more than one mechanism may be involved (16,17,18). 

In this study, bilateral diffuse mild or severe degree of renal uptake was observed in 43.5 % of adults and in one adolescent patient. Bilateral diffuse increased renal cortical uptake of 99mTc HDP is rare; it is present in only about 1% or less of bone scintigrams (17,27,28). It has been reported in cases of cirrhosis, lung cancer, thyroid cancer, primary hepatoma, Hodgkin’s disease, leukemia, sideroblastic anemia and diabetes mellitus (28). Renal vascular disease and iron overload was considered to be the major causes of this finding. Bernard et al reported that intense renal parenchymal uptake related to chemotherapy, radiotherapy or hypercalcemia was presented in six (%1) patients (27). Uptake of bone scanning agent by renal cortex was reported in a patient with advanced hepatic disease and oliguria (29). Increased serum iron levels was also found occasionally in cirrhotic patients. Renal blood flow was often reduced in cirrhotic paitients, even though their blood urea levels were normal. Patients with hepatorenal syndrome are characterized by severe cirrhosis, glomerular hypofiltration and low arterial pressure (30). Renal failure is a common major complication in patients with advanced cirrhosis and generally indicates a poor prognosis when combined with liver failure (30). In 50% of our adults with increased renal uptake, BUN values were found high. Patients with severe degree of renal uptake had higher serum BUN and creatinine values compared with patients without renal uptake. In addition, a significant difference existed in serum creatinine values between patients with mild and severe degree of renal uptake. Therefore, in patients with normal serum creatinine, diffuse increased renal cortical 99mTc HDP uptake may be correlated with a higher risk of subsequent deterioration in renal function. Consequently, increased renal uptake on BS may be an early marker of renal dysfunction or a predictor of hepatorenal syndrome. 

Diffuse liver uptake of bone tracer has been reported in cases of diffuse liver damage due to a variety of etiologies including massive hepatic necrosis associated with iron therapy, Budd-Chiari syndrome, cocaine hepatotoxicity, acute hypoxic hepatitis, ischemic hepatopathy, and high-dose methotrexate chemotherapy (31,32,33,34,35). Hepatic and other ectopic soft tissue calcifications have also been reported following LT (36). 

Awareness of these characteristic osseous and nonosseous scintigraphic findings may facilitate an accurate diagnosis and lead to more appropriate patient management. Bone scans may be also be used to predict progression in patients with CLD.

In the current study the osseous and nonosseous BS findings in LT candidates with end-stage CLD was presented. Because of the retrospective nature of study, we were unable to compare our results with bone mineral density (BMD) and all biochemical markers of bone metabolism and/or hormonal parameters. Further prospective investigations with larger patient groups are needed to clarify the significance and diagnostic value of the osseous and nonosseous finding in these patients. 

## CONCLUSION

The results of the current study haves shown that adolescent LT candidates with end-stage CLD had no osseous abnormality on BS. However, all adult patients exhibit one or more osseous abnormalities. Typical scintigraphic findings denoting to abnormalities in bone metabolism were generalized decreased osseous uptake, decreased lower extremity osseous uptake, increased periarticular uptake, increased cortical/periosteal uptake indicating hepatic HOA, and other unusual uptake patterns. Hepatocellular carcinoma metastases may present itself as rib metastases linear in pattern. Soft-tissue accumulation in the form of diffuse bilateral mild or severe degree of renal uptake and less commonly mild diffuse liver uptake can be observed. Increased renal uptake may be an early marker of renal dysfunction or a predictor of hepatorenal syndrome.

**Conflicts of Interest**

There are no conflicts of interest.

## Figures and Tables

**Table 1:**
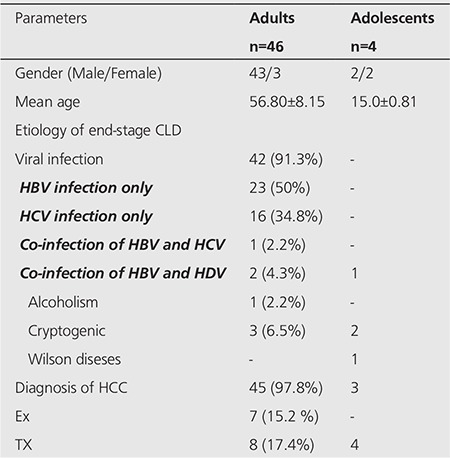
Patient characteristics

**Table 2:**
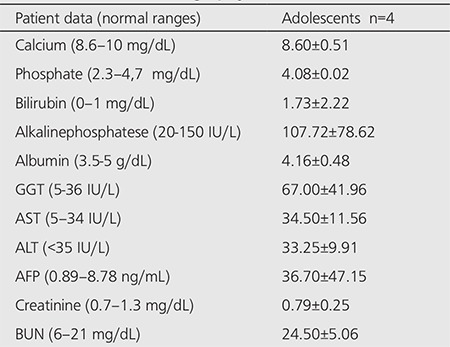
Biochemical finding in adolescent patients atthe time of bone scintigraphy

**Table 3:**
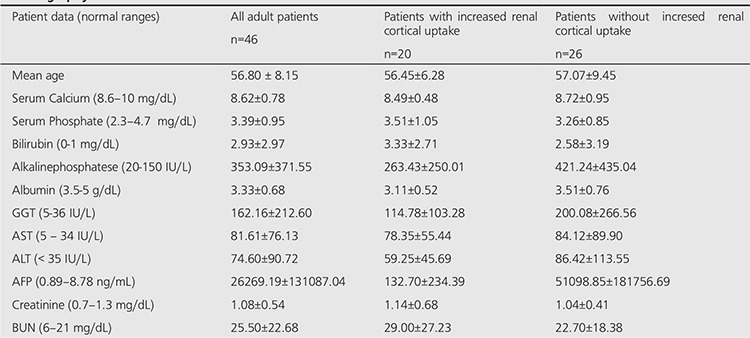
Biochemical finding in adult patients with and without increased renal cortical uptake at the time of bone scintigraphy

**Table 4:**
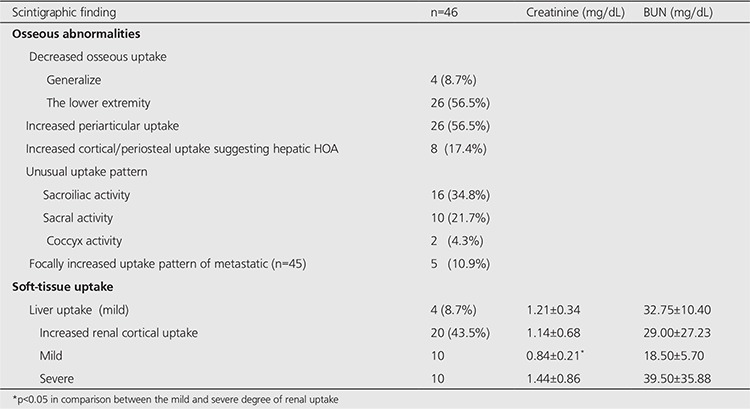
Osseous and nonosseous scintigraphic finding in adult LT candidates with end-stage CLD
